# Comparison of Different Green Extraction Techniques Used for the Extraction of Targeted Flavonoids from Edible Feijoa (*Acca sellowiana* (O.Berg) Burret) Flowers

**DOI:** 10.3390/plants12071461

**Published:** 2023-03-27

**Authors:** Katarzyna Angelika Gil, Stela Jokić, Ana-Marija Cikoš, Marija Banožić, Martina Jakovljević Kovač, Antonella Fais, Carlo Ignazio Giovanni Tuberoso

**Affiliations:** 1 Department of Life and Environmental Sciences, University of Cagliari, Cittadella Universitaria di Monserrato, S.P. Monserrato-Sestu km 0.700, 09042 Monserrato, Italy; 2 Faculty of Food Technology, Josip Juraj Strossmayer University of Osijek, Franje Kuhača 18, 31000 Osijek, Croatia; 3 Faculty of Agriculture and Food Technology, University of Mostar, Biskupa Čule bb, 88000 Mostar, Bosnia and Herzegovina

**Keywords:** feijoa flowers, green extraction, antioxidant activity, bioactive compounds, HPLC-PDA

## Abstract

This study aimed to investigate the effect of four green extraction techniques (ultrasound-assisted extraction, UAE; supercritical fluid extraction, SFE; subcritical water extraction, SWE; and extraction using deep eutectic solvents, DES) on the extraction of targeted flavonoids from edible feijoa flowers. The bioactive components in the obtained extracts were quantified by High-Performance Liquid Chromatography—Photodiode Array Detector (HPLC-PDA). Moreover, total polyphenol content and antioxidant activity by DPPH^•^, ABTS^•+^, FRAP, and CUPRAC assays were investigated. UAE generally gave the highest yields for isoquercitrin and quercetin content (18.36–25.33 and 10.86–16.13 µg/g), while DES extraction with choline chloride:lactic acid (1:2) and H_2_O content of 50% gave the highest yield of chrysanthemin (90.81 µg/g). The highest yield of flavone (12.69 mg/g) was obtained with supercritical CO_2_ at 300 bar. Finally, UAE gave the highest total polyphenol content (ca. 64 mg GAE/g) and antioxidant activity at 70 °C during 30 min with 40% (0.84 mmol TEAC/g and 2.25 mmol Fe^2+^/g, for ABTS^•+^ and CUPRAC, respectively) and 60% ethanol-water solution (0.49 mmol TEAC/g and 2.09 mmol Fe^2+^/g, for DPPH^•^ and FRAP, respectively). The eco-friendly extraction techniques resulted in selective methods capable of extracting targeted bioactive compounds from edible feijoa flowers.

## 1. Introduction

Feijoa (*Acca sellowiana* (O.Berg) Burret) belongs to the family Myrtaceae, and it is native to South America but now is cultivated worldwide. The plant is well known for its aromatic fruits [1], which have been widely investigated due to their beneficial impact on human health [1,2,3]. Recent research shows that there is a potential to develop new food products with feijoa using the fruit, flower, or leaves [4,5,6,7]. Since feijoa flowers (FF) are edible, they have gained a lot of attention from the scientific community due to their content of ascorbic acid and phenolic compounds [5,8], such as flavone, hyperoside, apigenin, and cyanidin-3-*O*-glucoside [7,8,9]. Flowers, due to the presence of bioactive compounds, can be a potential source of raw material for further processing into nutraceuticals [10,11]. For this reason, researchers are increasingly investigating flowers as a source of polyphenols with substantial bioactive potential, especially for the prevention and treatment of diseases caused by oxidative stress [12,13,14].

Extraction is a fundamental step in the path of phytochemical processing for the discovery, separation, and recovery of bioactive components from plant materials [15]. It can help the food industry develop new health ingredients, making the nutraceutical products more attractive and desirable to consumers. Nowadays, green extraction technologies (GETs) are starting to be regarded as cheap (with the exception of supercritical fluid extraction), fast, and eco-friendly procedures for obtaining bioactive constituents due to the minimization of energy and organic solvent consumption, as well as sample degradation, without being harmful to the environment [16,17,18,19]. The development of modern GETs, such as ultrasound-assisted extraction (UAE), supercritical fluid extraction (SFE), subcritical water extraction (SWE), and extraction using deep eutectic solvents (DESs), has been quickly and constantly increasing worldwide because they have significant advantages over conventional methods. UAE was introduced in the 1950s to improve hop flower extraction in beer processing [20] and has also been studied as a method for bioactive compound detection in flowers [21,22]. Water in a subcritical state has become a green solvent used for SWE and applied for biomolecule extraction from flowers and their residues [23,24]. SFE, one of the first green techniques used to extract high-value bioactive compounds such as caffeine from vegetal materials, has also been applied to flower material [25]. Finally, DESs have attracted scientists in recent years due to the low price of raw materials and easy preparation of the solvent, as well as their tunable properties, low toxicity, and biodegradability [26]. These solvents have become very efficient extraction techniques for obtaining valuable phenolic compounds, including moderately polar flavonoids, from a variety of plant materials [27], as well as flowers [28].

The present study aimed to compare the effects of four different GETs (UAE, SFE, SWE, and DESs) on five targeted flavonoids (hyperoside, isoquercitrin, quercetin, chrysanthemin, and flavone) content in FF. Moreover, the total polyphenol content (TPC) and antioxidant activity (AA) determined by DPPH^•^ (2,2′-diphenyl-1-picrylhydrazyl radical), ABTS^•+^ (2,2′-azinobis-(3-ethylbenzothiazoline-6-sulphonic acid)), FRAP (ferric reducing antioxidant power), and CUPRAC (cupric ion-reducing antioxidant capacity) assays were evaluated. To the best of our knowledge, no report has yet appeared on the comparison of GETs of *A. sellowiana* flower flavonoid compounds.

## 2. Results

### 2.1. Influence of Extraction Technique on Selected Phenolic Compounds Content in FF

**Figure 1:**
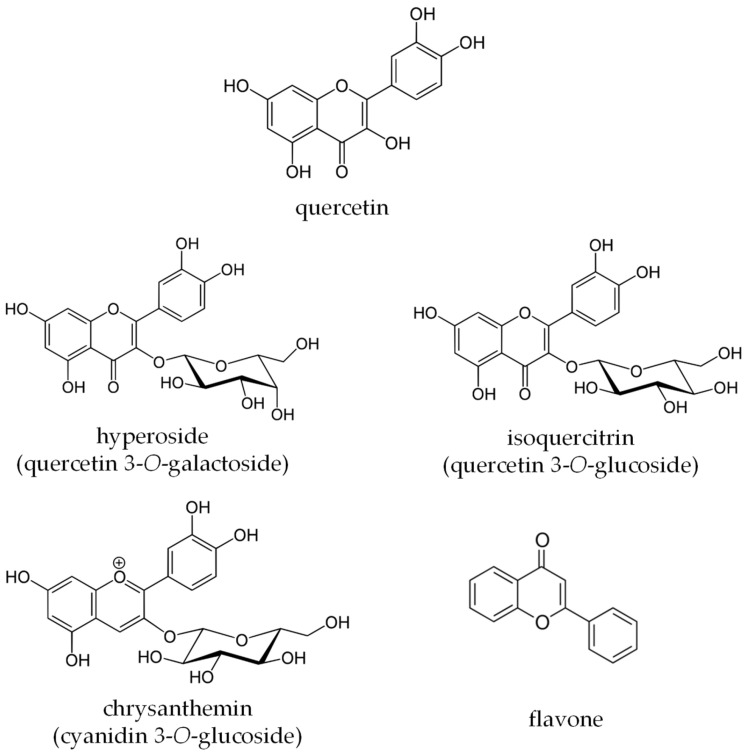
Targeted flavonoid compounds that were investigated in the feijoa flowers’ green extracts.

**Figure 2:**
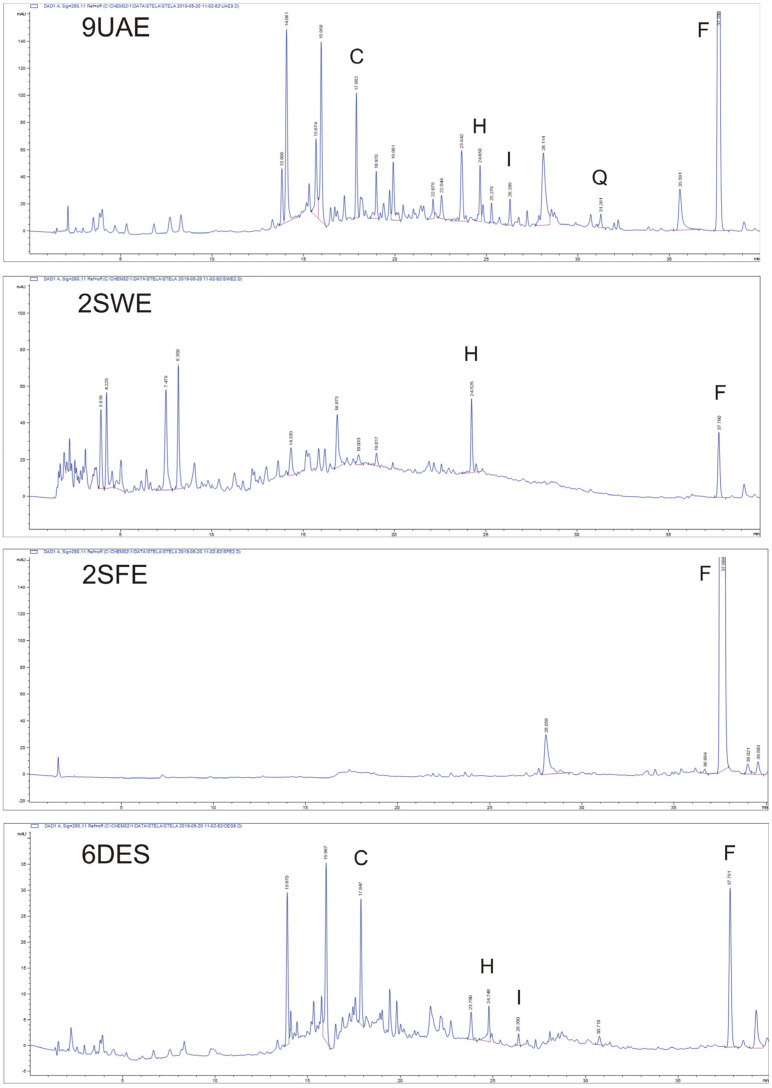
HPLC-PDA fingerprinting for selected samples of the four green extraction techniques (UAE: ultrasound-assisted extraction; SFE: supercritical fluid extraction; SWE: subcritical water extraction; DES: deep eutectic solvents) at λ = 280 nm. C: chrysanthemin; H: hyperoside; I: isoquercitrin; Q: quercetin; F: flavone. Chromatographic conditions are described in the text.

The four different GETs were selected taking into account the different physical-chemical mechanisms involved in the bioactive compounds’ separation [19]. Table 1 reports the different extraction conditions that were applied to extract the targeted flavonoids from edible FF. The five analysed targeted flavonoid compounds were three flavonols (quercetin, hyperoside, and isoquercitrin), one anthocyanin (chrysanthemin), and one flavone (Figure 1). These compounds were selected for both their significant amounts in the FF and their different polar properties. The applied HPLC-PDA method allowed us to easily detect the five flavonoids at 280, 360, and 520 nm, and Table 2 reports data in µg of pure compound per g of extract dry mass (dm). Figure 2 reports the general HPLC-PDA fingerprinting at λ = 280 nm of the four different GETs and the five targeted flavonoids detected in the feijoa flowers.

UAE generally showed to be the most effective GET to extract all five targeted compounds compared to the other techniques. The three flavonols, namely hyperoside (quercetin 3-*O*-galactoside), isoquercitrin (quercetin 3-*O*-glucoside), and quercetin, were detected at 8.47–12.28, 18.36–25.33, and 10.86–16.13 µg/g dm, respectively. Flavone (2-phenylchromone) showed a broader variation range according to the temperature used in the UAE, and the highest temperature (70 °C) allowed to extract ca. 2–3 times more than 30 °C, reaching yields ranging from 114.02 to 183.69 µg/g dm. Generally, a similar trend was observed in the case of the other four investigated compounds, providing more efficient flavone extraction with the use of higher temperatures.

SFE was demonstrated to be a very selective method, best for the extraction of flavone from FF but not able to extract the other four investigated compounds. Furthermore, the effect of the pressure played an important role in extraction yield: the higher pressure (300 bar, 1SFE) allowed the extraction of 12.69 mg/g dm of flavone, which accounts for 3 times more than 2SFE extraction.

SWE resulted in being less efficient than GET regarding the qualitative and quantitative extraction of targeted polyphenols from FF. Only hyperoside and flavone were extracted, and the latter in the lowest amount compared with the other three extraction techniques. Interestingly, this method showed to be better than DES and UAE in the extraction of hyperoside when a higher temperature (180 °C) was applied (20.33 µg/g dm, 2SWE).

Finally, DES generally showed no particular positive aspects in flavonoid extractions, as no quercetin was extracted and the amount of the other four targeted compounds was lower than that of other investigated GETs. In turn, this method showed to be comparable to that in the UAE regarding chrysanthemin (cyanidin 3-*O*-glucoside) extraction, with sample 6DES reaching a yield of 90.81 µg/g dm. Furthermore, this sample obtained with choline chloride:lactic acid (1:2) at 50% H_2_O content was the most suitable DES also for the extraction of hyperoside, isoquercitrin, and flavone.

### 2.2. Influence of Extraction Technique on Antioxidant Activity and Total Phenols in FF

The TPC and AA of obtained extracts were measured by cupric-reducing antioxidant activity (CUPRAC), ferric-reducing/antioxidant power (FRAP), and free radical-scavenging activity (DPPH^•^ and ABTS^•+^) assays (Table 3). The results of all performed assays showed approximately the same trends among examined extracts, showing a positive correlation between TPC measured by Folin-Ciocalteu’s assay and four antioxidant assays (Pearson correlation coefficients (*p* ≤ 0.05) of 0.9105, 0.9489, 0.9255, and 0.9000 for FRAP, CUPRAC, ABTS^•+^, and DPPH^•^, respectively).

Generally, the highest AA value (0.30–0.49 mmol TEAC/g of residue and 1.46–2.09 mmol Fe^2+^/g dm, for DPPH^•^ and FRAP, respectively) and TPC (53.20–65.56 mg GAE/g dm) were detected in UAE extracts using 40% and 60% ethanol-water solutions. Furthermore, the conditions of extraction 3UAE (70 °C, 40% EtOH, 30 min) and 6UAE (70 °C, 60% EtOH, 30 min) showed to be the most valuable from the TPC and AA points of view. A general increment in AA and TPC along with an increment in the temperature was also observed.

The extracts obtained using SWE were also rich in polyphenols (42.88 and 58.39 mg GAE/g dm, for 1SWE and 2SWE, respectively) and characterized by high AA (0.40 mmol TEAC/g dm, 0.98–1.13, and 1.24–1.39 mmol Fe^2+^/g dm, for ABTS^•+^, FRAP, and CUPRAC, respectively). Moreover, comparing two obtained SWE extracts, higher AA in the extract obtained using a higher temperature (180 °C; 2SWE) was observed. In contrast, the lowest TPC (2.87–3.90 mg GAE/g dm) and AA (0.01–0.04 mmol TEAC/g dm, for DPPH^•^ and ABTS^•+^, and 0.03–0.13 mmol Fe^2+^/g dm, for FRAP and CUPRAC) were found in both supercritical CO_2_ extracts.

Finally, DESs extracts showed moderate TPC and AA. The values for TPC for these extracts ranged from 25.93 to 51.51 mg GAE/g dm, while AA was in a range of 0.08–0.37 and 0.14–0.55 mmol TEAC/g dm (DPPH^•^ and ABTS^•+^, respectively), and 0.36–1.51 and 0.47–1.68 mmol Fe^2+^/g dm (FRAP and CUPRAC, respectively). As seen in the HPLC-PDA analysis, the DESs extract showing the best AA was 6DES obtained with choline chloride, lactic acid (molar ratio of 1:2), and 50% of water (*v*/*v*).

## 3. Discussion

### 3.1. Influence of Extraction Technique on Selected Phenolic Compounds Content in FF

As a result of the data in Table 2, UAE is a good extraction technique to extract all five targeted compounds, especially at higher temperatures. Our findings confirmed what had previously been observed by Benarfa et al. [29], who investigated UAE extracts of *Deverra scoparia* Coss. and Durieu flowers using response surface methodology. This scientific group confirmed that increasing the extraction temperature enhances the extraction efficiency by facilitating the distribution of the matrix content into the solvent after softening and breaking down the cell walls of the plant. The above can also be seen in the example of ultrasound-assisted extraction of flavonoids, including among others hyperoside and quercetin, from *Hypericum formosanum* with optimal extraction conditions such as ethanol concentration, 73.5%; extraction time, 38.3 min; and extraction temperature of 62.5 °C [30]. Similar results were observed in the extraction of quercetin from *Dendrobium officinale*, where the optimum conditions for UAE were an ethanol concentration of 81.6%, an extraction time of 30 min, and a temperature of 60 °C. With an additional increase in temperature up to 70 °C, there is a decrease in the quercetin extraction ratio, possibly due to the destruction or volatilization of quercetin [31]. In our case, the extraction took place at a maximum temperature of 70 °C, at which no decomposition of quercetin was observed, i.e., an increase in the amount of quercetin compared to 50 °C was observed. In the case of hyperoside extraction from *Crataegus monogyna* Jacq. (hawthorn), among the extraction techniques used such as Soxhlet, maceration, UAE, and microwave-assisted extraction (MAE), UAE using 50% ethanol for 30 min at room temperature proved to be the most suitable [32].

The amount of flavonoids extracted from FF by SWE was less interesting, but with this GET, it was also observed that higher temperatures increased the extraction yield. The influence of temperature on extraction yield efficiency is because water has a lower relative permittivity at a higher temperature, which enfeebles the hydrogen bonds and makes subcritical water more comparable to less-polar organic solvents (ethanol and methanol). Thus, the solubility of less polar phenolic compounds increases with the increment in subcritical water temperature [33]. The same tendency was noticed in extracting pomegranate peel, where a significant increase in the polyphenol content was observed with the rise in temperature from 100 to 160 °C [34]. Nevertheless, the further increase in temperature had a negative influence on the polyphenol content, probably due to the degradation of antioxidants at higher temperatures. In addition, according to the work of Ko et al. [35], the efficiency of SWE of flavonoids is significantly affected not only by the extraction conditions but also by the structure of the flavonoids themselves, especially in the case of the presence of side chains, glucose, and double bonds, and the solubility of the components itself is affected by their ability to generate hydrogen bonds with the solvent. Despite the fact that quercetin and isoquercitrin were extracted at temperatures of 110 and 150 °C and in a time of 5 and 15 min in the mentioned work, in our work the mentioned components were not extracted. By comparing UAE and SWE as a modern technique of extracting components from *Arctostaphylos uva-ursi* L. herbal dust with conventional solid–liquid extraction, it was shown that a significantly high content of hyperoside in the extract was accomplished with UAE. The highest content of hyperoside was achieved using 70% ethanol at 40 °C for 40 min. In addition, certain amounts of hyperoside were also shown using SWE at 178.5 °C for 12 min with 0.05% HCl, which is similar to the conditions used in this work. However, contrary our results, where a higher proportion of hyperoside compared to UAE was achieved under SWE conditions of 180 °C and 15 min, in the work of Živković et al. [36] the situation is reversed.

In the case of SFE, pressure is a key factor, and higher values allow for better recoveries. This confirms what was previously observed [37,38,39]: that the increment of the extraction yield correlates with the increment of the pressure due to the increased density and solvating power of CO_2_ in supercritical condition. In addition, the extraction temperature is also important, considering that these two parameters play an important role in the solubility of solutes in the solvent [40]. In the extraction of flavonoid compounds from *Xinjiang jujube* (*Ziziphus jujuba* Mill.) using SFE, UAE, and conventional Soxhlet extraction (CSE), it is noticeable that the extracts obtained using SFE-CO_2_ under optimal conditions had a higher concentration of total flavonoids as well as most individual ones, such as hyperoside and quercetin glucoside. The obtained optimal conditions for SFE extraction of flavonoids are a temperature of 52.52 °C, a pressure of 27.12 MPa, a time of 113.42 min, and a cosolvent flow rate of 0.44 mL/min. Despite the similar pressure as in our 2SFE sample, the SFE extracts obtained from edible feijoa flowers do not contain the mentioned components, which speaks in favor of the importance of extraction and optimization of each plant material for extraction [41].

A comparison of literature data with the DESs mixture used in this research is not easy due to the different molecules involved in the studies. Although the use of DES in the extraction of flavonoids has yielded more than promising results, there is still a problem with the selectivity of DES/NADES in the extraction of individual flavonoids. Most of the published papers on the topic of flavonoid extraction using DESs [42] show the results obtained as total flavonoids or antioxidant activity, while the content of individual flavonoids with different DESs and under different extraction conditions has not been examined sufficiently. Additionally, the DESs mixture of choline chloride: lactic acid (molar ratio 1:2), and 50% water (*v*/*v*) showed to be the best for the extraction of rutin from *Satureja montana* L. [26], confirming that the water content in the DES mixture has an important influence on the extraction efficiency, as well as temperature and time of extraction. For the extraction of flavonoids from the rhizomes of *Polygonatum odoratum*, the most suitable solvent turned out to be choline chloride: lactic acid (1:2), which coincides with our results [43].

Selectivity in flavonoid extraction is fundamental for preparing extracts enriched with high-value compounds. For instance, flavone was identified in all extracts, but SFE is highly selective in extracting this hydrophobic compound. Taking into account that this molecule shows antioxidant, anti-estrogenic, and antileishmanial properties [44] and can be used for producing semisynthetic derivatives, this GET is very interesting. Chrysanthemin and isoquercitrin were identified in UAE and DES extracts. Chrysanthemin is the main anthocyanin present in edible flower petals and shows anti-inflammatory potential [14]. Hyperoside was found in UAE, SWE, and DES extracts, but SWE at 180 °C improved its extraction. Finally, quercetin was found only in UAE extracts, resulting in a very selective technique for this flavonol. Quercetin and its glycosides show interesting pharmacological properties such as antioxidant, anti-inflammatory, anti-tumor, and anti-cardiovascular disease activities [45].

### 3.2. Influence of Extraction Technique on Antioxidant Activity and Total Phenols in FF

The positive correlation between the TPC and the four antioxidant assays observed in the FF extracts obtained with the GETs was also reported by Pinedo-Espinoza et al. [13]. Moreover, a general increment in AA and TPC along with an increment in the temperature was also observed. This confirmed Benarfa et al.’s [29] results on the effect of the extraction period, temperature, and sample-to-methanol ratio on the AA of *D. scoparia* flower extracts. This was observed in SWE by Jokić et al. [46], and also Nastić et al. [47] reported this trend, showing that the increase of the extraction temperature from 60 °C to 160 °C influenced the increment of AA as well as for TPC.

### 3.3. Comparison of Results with Available Literature

By reviewing the literature, we found only several papers on the extraction of components from feijoa flowers and antioxidant activity, but as far as we know, there is no paper comparing different extraction techniques on the amount of components and antioxidant activity of flower extract. In a study by Montoro et al. [7], it was shown that the feijoa flower extract obtained using 80% ethanol in an ultrasonic bath at 10 °C shows a high amount of TPC (395.14 ± 7.91 mg GAE/L) and good antioxidant activity measured by several methods. Comparing our results with the obtained ones, we can see that the obtained results are much higher in terms of TPC and antioxidant activity. In addition, the authors examined the composition of the obtained extract using chromatographic techniques, which confirmed the presence of quercetin (2.6 ± 0.0 mg/L), among other components. It is certainly important to note that TPC is also affected by the flowering and harvesting stages, as was proven in the work of Magri et al. [5], where, depending on the time of picking, TPC amounted to 51.42–113.4 mg GAE/100 g of FW, with the highest values in the case when the petals are fully open and the anthers, filaments, and carpels have a dark reddish color. In the work of Aoyama et al. [9], the polyphenolic composition of different parts of the plant, including flower buds from the plant *Feijoa sellowiana,* was investigated. Among the tested components, hyperoside (30.2 ± 0.2 µg/10 mg extract) and flavone (56.3 ± 2.8 µg/10 mg extract) were also quantified in the methanolic and purified flower buds’ extract.

## 4. Materials and Methods

### 4.1. Chemicals

All solvents used for the extraction procedures were of analytical grade and purchased from J.T. Baker (PA, USA). The CO_2_ that was used for SFE extraction was 99.97% (*w*/*w*) pure and obtained from Messer (Osijek, Croatia). Standard flavonoids were purchased from Extrasynthese (Genay Cedex, France). The Folin-Ciocalteu reagent as well as 2,2-diphenyl-1-picrylhydrazyl radical (DPPH^•^) and gallic acid were purchased from Sigma-Aldrich (USA). Ferrous sulphate, CuCl_2_·2H_2_O, ammonium acetate, neocuproine (2,9-dimethyl-1,10-phenanthroline) hydrochloride, (±)-6-hydroxy-2,5,7,8-tetramethylchroman-2-carboxylic acid (Trolox), 2,2′-azino-bis(3-ethylbenzothiazoline-6-sulfonate radical cation (ABTS^•+^), potassium persulphate, acetic acid, ferric chloride, CuSO4·5H_2_O, 2,4,6-tris(2-pyridyl)-1,3,5-triazine (TPTZ), CH_3_COONa·3H_2_O were obtained from Merck-Sigma-Aldrich (Milan, Italy).

### 4.2. Plant Material

*Acca sellowiana* (O.Berg) Burret mature flowers (FF) from cultivated, growing plants were randomly harvested in June 2021 in an experimental field in Uta (Uta, Italy; 39°23′58″ N 9°19′93″ E) by professional pickers. The specimens were identified by Prof. Andrea Maxia (University of Cagliari, Italy). After the collection, the flowers were cleaned, frozen, and freeze-dried (Lio 5 POGT, Trezzano, Italy). Before extractions, FF was homogenized and ground using an IKA M 20 laboratory mill (IKA, Staufen, Germany) to obtain a powder sample. The extract dry mass (dm) was evaluated in triplicate by drying the solution (500 μL) for 5 h in a thermostatic oven at 105 ± 1 °C to a constant weight.

### 4.3. Extraction Techniques

#### 4.3.1. Ultrasound-Assisted Extraction (UAE)

The extractions were performed according to the protocol described previously by Banožić et al. [48]. In addition, 1 g of powdered FF samples were placed in 10 mL of three different aqueous ethanol solutions (40:60, 60:40, and 80:20, H_2_O:EtOH% *v*/*v*). Next, the samples were placed in an ultrasound bath with temperature control (30, 50, or 70 °C) at 37 kHz and an ultrasonic power of 50 W (Elma, Elmasonic P70H, Germany) for 30 min. The samples were filtered through a PTFE 0.45 μm filter before further analyses.

#### 4.3.2. Supercritical Fluid Extraction (SFE)

The extractions were performed in the SFE system, and the process had previously been described by Jokić et al. [49]. The powdered FF samples (100 g) were placed into an extractor vessel, and the extracts were collected in previously weighted glass tubes at 15 bar and 25 °C. The extractions were performed at a temperature of 40 °C for 90 min at two different pressures (100 and 300 bar), and the CO_2_ mass flow rate of 2 kg CO_2_/h was kept constant during the process.

#### 4.3.3. Subcritical Water Extraction (SWE)

The extraction was carried out in a handmade subcritical water extraction system described in detail by Jokić et al. [46]. The powdered FF sample (10 g/100 mL) was placed into a 500 mL extraction vessel made from stainless steel AISI 304. The extractions were performed at two different temperatures (130 °C and 180 °C) with a reaction time of 15 min at a working pressure of 30 bar. The Feijoa flower-water mixture was poured into the reactor. The extraction vessel was heated in an oven to the desired temperature (130–180 °C). The mixture was stirred with a magnetic stirrer placed below the extractor vessel to obtain adequate stirring of water and material. The N_2_ was used to control pressure and provide an inert state during the extractions. When the extraction was finished, the reactor was rapidly cooled in an ice bath. The reactor content was filtered through filter paper, and the water extracts were obtained.

#### 4.3.4. Extraction with Deep Eutectic Solvents (DESs)

The DESs mixtures were prepared according to Jokić et al. [50] by mixing choline chloride as a hydrogen bond acceptor (HBA) with 3 different hydrogen-bond donors (HBDs): urea, glycerol, or lactic acid in a molar ratio of 1:2. The DESs mixtures were stirred and heated at 80 °C (about one hour) till homogenous liquid states were obtained. Next, dried and milled FF (50 mg) was mixed with 1 mL of solvent: a DESs mixture with 10 or 50% (*v*/*v*) of ultrapure water (Millipore Simplicity 185, Darmstadt, Germany). The prepared samples were stirred at 1500 rpm in an aluminium block on a Stuart SHB magnetic stirrer at 50 °C for 1 h. After the extraction, the samples were centrifuged at 6000 rpm for 5 min and then decanted. The supernatant was used for further investigation.

### 4.4. Determination of Phenolic Compounds by HPLC

The qualitative and quantitative analyses of phenolic compounds were carried out using an HPLC-PDA method as described by Tuberoso et al. [51] with slight modifications. An HPLC Agilent 1260 Infinity II system was employed, fitted with a quaternary pump (G7111B), an autosampler (G7129A), and a diode array detector (G7117C), set at 280, 320, 360, and 520 nm. Separation was obtained with a Zorbax Eclipse Plus C18 (Agilent, USA) column (100 × 4.6 mm, 5 µm) using 0.2 M phosphoric acid (solvent A) and acetonitrile (solvent B) at a constant flow rate of 1.0 mL/min. The gradient (*v*/*v*) was generated, decreasing from 90% of solvent A to 85% in 1.40 min; to 65% in 12.63 min; to 50% in 21.05 min; and to 90% in 30 min. Before each injection, the system was stabilized for 10 min with the initial A/B ratio (90:10, *v*/*v*). The injection volume was 6.70 μL. Chromatograms and spectra were elaborated with the OpenLab V. 2.51 data system (Agilent Technologies, Cernusco sul Naviglio, MI, Italy). Anthocyanins were detected and dosed at 520 nm, quercetin derivatives at 360 nm, and flavone at 280 nm. Standard solutions were prepared in methanol, filtered through an Econofilter RC membrane (0.45 μm, Ø 25 mm, Agilent Technologies, Milan, Italy), and injected into HPLC without any further purification. Hyperoside, isoquercitrin, quercetin, chrysanthemin (cyanidin-3-*O*-glucoside), and flavone were dosed using their calibration curves, built with the method of the external standard, correlating the area of the peaks vs. the concentration. The correlation values were 0.99847–0.99995 in the range of 20–200 ppm.

### 4.5. Determination of Total Polyphenol Content (Folin–Ciocalteu’s Assay), Free Radical Scavenging Activity (ABTS^•+^ and DPPH^•^ Assays), and Total Reducing Power (CUPRAC and FRAP Assays)

All assays were carried out on a Cary 50 spectrophotometer (Varian, Leinì, TO, Italy) using 10 mm Kartell^®^ plastic cuvettes. Sample solutions were prepared by dissolving 10 mg of the extract dry mass into 1 mL of methanol. Working solutions were properly diluted with MeOH in the range of 1:1–1:100 *v*/*v* before the analysis to fit with the calibration curve ranges. Total polyphenol content (TPC) was measured by the modified Folin-Ciocalteu spectrophotometric method [51,52]. Briefly, 100 µL of the diluted sample was mixed with 500 µL Folin-Ciocalteu reagent, and then, after 5 min, 3 mL of 10% Na_2_CO_3_ (*w*/*v*) was added. The mixture was agitated, diluted with water to a final volume of 10 mL, and then left for a 90 min incubation period at room temperature. The absorbance was read at 725 nm against a blank. The TPC results were expressed as mg of gallic acid equivalent (GAE) per g of residue, using a calibration curve of a freshly prepared gallic acid standard solution (10–200 mg/L). The DPPH^•^ was performed according to Tuberoso et al. [52]. A total of 50 µL of diluted extract or standard was added to 10 mm cuvettes with 2 mL DPPH^•^ solution (0.04 mmol/L in methanol). The spectrophotometric readings were carried out at 517 nm after 60 min. The ABTS^•+^ assays were performed according to Re et al. [53], with some modifications [52]. The ABTS radical cation (ABTS^**•**+^) was produced by reacting ABTS stock solution with 70 mM potassium persulfate (final concentration), and the mixture was allowed to stand in the dark at room temperature for 12–16 h before use. After this time, 4 mL of the reaction mixture were diluted with water, and a 0.08 mM ABTS^**•**+^ solution was obtained. A total of 20 µL of the diluted extract or the standard was added to 10 mm cuvettes with 2 mL of 0.08 mM ABTS^**•**+^ solution and mixed. The spectrophotometric readings were carried out at 734 nm immediately after sample preparation. For the quantitative analysis of both DPPH^•^ and ABTS^•+^ assays, a calibration curve in the range of 0.02–1.0 mmol/L was prepared for Trolox, and data were reported as Trolox equivalent antioxidant capacity (mmol TEAC/g of residue). The FRAP assay was assessed by preparing a ferric complex of 2,4,6-tris(pyridin-2-yl)-1,3,5-triazine (TPTZ) and Fe^3+^ according to Bouzabata et al. [52]. Additionally, two mL of freshly prepared reagent (0.3123 g TPTZ and 0.5406 g FeCl_3_·6H_2_O in 100 mL acetate buffer = pH 3.6) were added to 20 µL of the diluted extract solution or the standard in 10 mm cuvettes. The spectrophotometric readings were carried out at λ = 593 nm after 60 min. The CUPRAC assay was performed according to Bektaşǒglu et al. [54], with some modifications [52]. A total of 1 mL water, 500 µL copper (II) chloride, 500 µL neocuproine, 500 µL ammonium acetate, and 100 µL methanol (blank), standard, or sample were added to 10 mm polystyrene cuvettes in that order, and the spectrophotometric readings were carried out at λ = 450 nm after 30 min. Quantitative analysis of both FRAP and CUPRAC assays was done using the external standard method, employing ferrous sulfate in the range of 0.1–2 mmol and expressed as mmol Fe^2+^/g of residue.

### 4.6. Statistical Analysis

All measurements were performed in triplicate, and results were expressed as means ± standard deviation, with the significance level set at *p* ≤ 0.05.

## 5. Conclusions

The obtained results showed that different selected GETs have a divergent influence on the yield of targeted flavonoids from edible FF, as well as on the AA and TPC of obtained extracts. The results indicate that extraction mostly depends on solvent polarity as well as on the applied extraction parameters. Thus, generally, the highest yields of hyperoside, isoquercitrin, quercetin, and chrysanthemin were obtained by the UAE technique, but the DES extraction with choline chloride:lactic acid (1:2) and H_2_O content of 50% provided the highest chrysanthemin yield. Moreover, FF extracts obtained by the SFE technique showed the presence of flavone in the highest amount, especially under the pressure of 300 bars, compared with other GETs. Finally, the highest AA and TPC were detected in UAE extracts using a 40% and 60% ethanol-water solution, respectively. To sum up, flavonoids present in *A. sellowiana* flowers can be extracted in a preferential manner according to the extraction technique. Thus, the FF, thanks to the bioactive components, is an interesting and desirable raw material that can be further processed for the pharmaceutical, cosmetic, and food industries.

## Figures and Tables

**Table 1:** Feijoa flower samples and parameters of the green extraction techniques used.

Sample Code	Extraction Techniques	Extraction Parameters		
		Temperature (°C)	Time (min)	Solvent
1UAE	ultrasound-assisted extraction	30	30	H_2_O-EtOH 60–40% *v*/*v*
2UAE	ultrasound-assisted extraction	50		
3UAE	ultrasound-assisted extraction	70		
4UAE	ultrasound-assisted extraction	30		H_2_O-EtOH 40–60% *v*/*v*
5UAE	ultrasound-assisted extraction	50		
6UAE	ultrasound-assisted extraction	70		
7UAE	ultrasound-assisted extraction	30		H_2_O-EtOH 80–20% *v*/*v*
8UAE	ultrasound-assisted extraction	50		
9UAE	ultrasound-assisted extraction	70		
		temperature (°C)	time (min)	pressure (bar)
1SFE	supercritical fluid extraction	40	90	300
2SFE	supercritical fluid extraction	40	90	100
1SWE	subcritical water extraction	130	15	30
2SWE	subcritical water extraction	180	15	30
		Extraction solvent		
1DES	deep eutectic solvents	Choline chloride:urea 1:2; H_2_O content 10%		
2DES	deep eutectic solvents	Choline chloride:urea 1:2; H_2_O content 50%		
3DES	deep eutectic solvents	Choline chloride:glycerol 1:2; H_2_O content 10%		
4DES	deep eutectic solvents	Choline chloride:glycerol 1:2; H_2_O content 50%		
5DES	deep eutectic solvents	Choline chloride:lactic acid 1:2; H_2_O content 10%		
6DES	deep eutectic solvents	Choline chloride:lactic acid 1:2; H_2_O content 50%		

**Table 2:** Concentrations of targeted flavonoids obtained from feijoa flowers extracted with different green extraction techniques.

Sample Code	Hyperoside (µg/g dm)	Isoquercitrin (µg/g dm)	Quercetin (µg/g dm)	Chrysanthemin (µg/g dm)	Flavone (µg/g dm)
1UAE	8.47 ± 0.42 ^fg^	19.26 ± 1.54 ^c^	10.86 ± 0.54 ^c^	58.00 ± 3.48 ^e^	42.44 ± 1.70 ^i^
2UAE	8.92 ± 0.45 ^ef^	18.90 ± 0.94 ^c^	11.32 ± 0.68 ^c^	58.19 ± 3.49 ^e^	55.53 ± 3.33 ^h^
3UAE	9.24 ± 0.37 ^def^	18.36 ± 0.92 ^c^	11.48 ± 0.92 ^c^	65.13 ± 5.21 ^cd^	114.02 ± 6.84 ^e^
4UAE	9.85 ±0.69 ^cde^	21.77 ± 1.09 ^b^	13.63 ± 0.68 ^b^	68.84 ± 5.51 ^bc^	57.73 ± 2.31 ^h^
5UAE	10.02 ± 0.50 ^cd^	21.68 ± 1.30 ^b^	13.39 ± 1.07 ^b^	71.67 ± 3.58 ^bc^	68.28 ± 4.10 ^g^
6UAE	10.58 ± 0.95 ^c^	21.53 ± 1.08 ^b^	13.02 ± 1.04 ^b^	73.38 ± 3.67 ^b^	135.29 ± 8.12 ^d^
7UAE	10.23 ± 0.51 ^cd^	23.88 ± 1.19 ^a^	15.54 ± 1.24 ^a^	68.39 ± 5.47 ^bc^	66.39 ± 3.98 ^g^
8UAE	10.77 ± 0.54 ^c^	23.74 ± 1.19 ^a^	15.16 ± 1.21 ^a^	70.78 ± 3.54 ^bc^	77.86 ± 4.67 ^f^
9UAE	12.28 ± 0.98 ^b^	25.33 ± 1.01 ^a^	16.13 ± 0.81 ^a^	69.29 ± 5.54 ^bc^	183.69 ± 14.69 ^c^
1SFE	nd	nd	nd	nd	12,686.25 ± 253.73 ^a^
2SFE	nd	nd	nd	nd	4175.51 ± 41.76 ^b^
1SWE	7.82 ± 0.39 ^gh^	nd	nd	nd	13.32 ± 0.80 ^o^
2SWE	20.33 ± 1.02 ^a^	nd	nd	nd	11.82 ± 0.71 ^o^
1DES	7.33 ± 0.59 ^hi^	16.55 ± 0.83 ^d^	nd	73.42 ± 2.20 ^b^	21.14 ± 1.27 ^n^
2DES	8.37 ± 0.42 ^fg^	18.40 ± 1.66 ^c^	nd	60.08 ± 2.40 ^de^	32.51 ± 1.95 ^l^
3DES	6.60 ± 0.46 ^i^	15.19 ± 1.22 ^d^	nd	72.02 ± 5.76 ^bc^	21.58 ± 0.86 ^n^
4DES	7.26 ± 0.36 ^hi^	16.27 ± 0.81 ^d^	nd	73.06 ± 1.46 ^b^	25.0 6± 1.50 ^m^
5DES	5.41 ± 0.27 ^j^	12.63 ± 0.63 ^e^	nd	54.11 ± 4.33 ^e^	23.01 ± 1.38 ^nm^
6DES	8.88 ± 0.27 ^ef^	20.10 ± 1.41 ^bc^	nd	90.81 ± 2.72 ^a^	58.34 ± 1.75 ^h^

Expressed per g of extracted dry mass (dm), mean ± standard deviation (*n* = 3). Mean values within a column with different letters are significantly different at *p* ≤ 0.05; nd: not detected.

**Table 3:** Antioxidant activity of feijoa flower extracts obtained with selected different green extraction techniques.

Sample Code	TPC ^A^	DPPH^• B^	ABTS^•+ B^	FRAP ^C^	CUPRAC ^C^
	(mg GAE/g dm)	(mmol TEAC/g dm)		(mmol Fe^2+^/g dm)	
1UAE	55.57 ± 2.58 ^cd^	0.38 ± 0.03 ^c^	0.71 ± 0.01 ^d^	1.60 ± 0.19 ^bc^	1.87 ± 0.02 ^d^
2UAE	59.83 ± 1.99 ^bc^	0.43 ± 0.02 ^b^	0.75 ± 0.03 ^c^	1.59 ± 0.07 ^bc^	2.09 ± 0.05 ^b^
3UAE	65.56 ± 3.07 ^a^	0.42 ± 0.03 ^b^	0.84 ± 0.00 ^a^	2.01 ± 0.49 ^a^	2.25 ± 0.07 ^a^
4UAE	60.42 ± 5.18 ^bc^	0.35 ± 0.04 ^c^	0.65 ± 0.02 ^e^	1.46 ± 0.13 ^c^	1.73 ± 0.02 ^e^
5UAE	53.20 ± 2.50 ^de^	0.30 ± 0.04 ^d^	0.69 ± 0.02 ^d^	1.89 ± 0.01 ^ab^	1.93 ± 0.06 ^c^
6UAE	63.07 ± 7.65 ^ab^	0.49 ± 0.03 ^a^	0.80 ± 0.03 ^b^	2.09 ± 0.34 ^a^	2.13 ± 0.06 ^b^
7UAE	46.44 ± 2.64 ^fg^	0.25 ± 0.01 ^ef^	0.42 ± 0.02 ^hi^	1.09 ± 0.13 ^de^	1.03 ± 0.02 ^i^
8UAE	34.90 ± 3.34 ^h^	0.27 ± 0.02 ^de^	0.44 ± 0.03 ^h^	1.16 ± 0.14 ^d^	1.12 ± 0.02 ^h^
9UAE	48.58 ± 0.79 ^ef^	0.36 ± 0.02 ^c^	0.52 ± 0.03 ^g^	1.63 ± 0.30 ^bc^	1.35 ± 0.01 ^f^
1SFE	2.87 ± 0.26 ^k^	0.02 ± 0.00 ^j^	0.03 ± 0.00 ^n^	0.13 ± 0.00 ^hi^	0.05 ± 0.00 ^m^
2SFE	3.90 ± 0.18 ^k^	0.01 ± 0.00 ^j^	0.04 ± 0.00 ^n^	0.06 ± 0.00 ^i^	0.03 ± 0.00 ^m^
1SWE	42.88 ± 1.93 ^g^	0.25 ± 0.02 ^ef^	0.40 ± 0.00 ^i^	0.98 ± 0.04 ^de^	1.24 ± 0.09 ^g^
2SWE	58.39 ± 0.27 ^bc^	0.21 ± 0.01 ^g^	0.40 ± 0.00 ^i^	1.13 ± 0.03 ^d^	1.39 ± 0.03 ^f^
1DES	31.42 ± 0.80 ^hi^	0.21 ± 0.01 ^g^	0.35 ± 0.01 ^j^	0.90 ± 0.07 ^de^	1.00 ± 0.01 ^ij^
2DES	26.12 ± 2.13 ^j^	0.08 ± 0.01 ^i^	0.14 ± 0.03 ^m^	0.36 ± 0.01 ^gh^	0.47 ± 0.01 ^l^
3DES	27.95 ± 0.94 ^ij^	0.23 ± 0.01 ^fg^	0.33 ± 0.00 ^j^	0.81 ± 0.12 ^ef^	0.96 ± 0.00 ^j^
4DES	25.93 ± 3.67 ^j^	0.16 ± 0.02 ^h^	0.23 ± 0.01 ^l^	0.59 ± 0.07 ^fg^	0.66 ± 0.01 ^k^
5DES	32.75 ± 1.15 ^hi^	0.23 ± 0.02 ^fg^	0.30 ± 0.01 ^k^	0.87 ± 0.02 ^def^	0.95 ± 0.02 ^j^
6DES	51.51 ± 0.55 ^de^	0.37 ± 0.02 ^c^	0.55 ± 0.00 ^f^	1.51 ± 0.10 ^c^	1.68 ± 0.01 ^e^

All values are expressed per g of extract dry mass (dm), mean ± SD (*n* = 3); mean values within a column with different letters are significantly different at *p* ≤ 0.05. ^A^: total phenolic content (TPC) value is expressed as milligrams of gallic acid equivalent (GAE) per gram of residue. ^B^: DPPH^•^ and ABTS^•+^ values is expressed as the millimolar concentration of TEAC, obtained from a Trolox solution having an antiradical capacity equivalent to that of the extract. ^C^: FRAP and CUPRAC values are expressed as the millimolar concentration of Fe^2+^, obtained from a dilution of FeSO_4_ having an equivalent antioxidant capacity to that of the extract.

## Data Availability

Date are content within this article.
